# Herbal Use among Presurgical Patients in Turkey: A Cross-Sectional Study

**DOI:** 10.1155/2018/1643607

**Published:** 2018-04-10

**Authors:** Fulya Yilmaz, Hazal Ezgi Çifci

**Affiliations:** Anaesthesiology and Reanimation Department, Bozyaka Training and Research Hospital, Health and Science University, İzmir, Turkey

## Abstract

**Introduction:**

For centuries before the advent of modern medicine, traditional medicinal plants were the major agents for primary health care. Their use declined in most developed western countries during the last century's industrialization and urbanization. But, the last two decades have witnessed a new resurgence of interest in herbal and alternative medicines.

**Methods:**

The survey was conducted at the Anaesthesiology and Reanimation Department of Training and Research Hospital of Health and Science University among patients who had undergone elective surgery between January 1st 2016 and April 1st 2016. A questionnaire composed of 15 questions was used.

**Results:**

A total 87 (14.5%) patients reported the use of herbal medications. Twenty five patients were taking a single herbal medication and 52 patients were taking more than one. 92.5% of patients do not know the side effects of herbal medications. 35 cases of operation patients were questioned by the physician about herbal use, and 228 cases were not questioned by the physician.

**Conclusion:**

Anesthesiologist should be aware of the effects of herbals on body functions and possible herbal-drug interactions to take care of such potentional perioperative complications.

## 1. Introduction

For centuries before the advent of modern medicine, traditional medicinal plants were the major agents for primary health care [1–3]. Their use however declined in most developed western countries during the last century's industrialization and urbanization [2]. But the last two decades have witnessed a new resurgence of interest in herbal and alternative medicines [1, 2, 4, 5]. According to the WHO, about 70% of the world population currently uses medicinal herbs as complementary or alternative medicine [2]. Previous studies reported that 14–16% of American adult population and 49.4% of Israeli population consume herbal supplements often concomitantly with medications [2].

Herb-drug interaction (HDI) is one of the most important clinical concerns with concomitant use of medicinal herbs with medical drugs [2]. Until recently, most trained physicians have lack of adequate knowledge on medicinal herbs and HDI. On the other hand, most patients do not consider that it is necessary to disclose their herbal consumptions to physicians [2].

The adverse effects of herbal medicines include: toxicity due to overdose, physiological changes on bodily systems, and adverse drug interactions. Potential problems of herbal medications can be broadly classified as coagulation disorders (bleeding, and anticoagulation), cardiovascular side effects (hypertension, oedema, and disturbance of cardiac rhythm), water and electrolyte disturbances (sodium retention, and hypokalaemia), endocrine effects (hyperglycaemia, and hypoglycaemia), hepatotoxicity, and prolongation of anaesthetic agents (confusion, and sedation) [1, 4–9]. The aim of this study was to investigate the use of herbal remedies in patients attending for anaesthesia before operation in our hospital.

## 2. Material and Methods

Our prospective, cross-sectional survey was conducted at the Anaesthesiology and Reanimation Department of Training and Research Hospital of Health and Science University among patients who had undergone elective surgery between January 1, 2016, and April 1, 2016. There was no special exclusion criterion. Ethical approval was obtained from the Training and Research Hospital of Health and Science University Local Ethics Committee. Written, informed consent of the participating patients was obtained. The questionnaire included sociodemographic characteristics (age, gender, and education), what kind of herbs the patient is using, and communication between the participants and their physicians about herbal use.

Analysis of data in the form of frequencies was conducted using SPSS 16.00 statistical software. Chi-square was used to characterize nominal variables. A *p* value <0.05 was considered statistically significant.

## 3. Results

During the 3-month period, 600 patients responded the questionnaire at preoperative evaluation before undergoing anaesthesia. A total of 87 (14.5%) patients reported the use of herbal medications. Twenty five patients were taking a single herbal medication, and 52 patients were taking more than one. Male patients (51 out of 337) used herbal medications more frequently than females (36 out of 263) (*p* > 0.05). The incidence of responders is 4% (*n*=24) under 18 years while 96% (*n*=576) over 18 years. The number of cases using herbal under 18 years is 3. This indicates that parents use herbal agents for their children. The most commonly used medications are garlic (*n*=19), fish oil (*n*=14), Swedish bitter (*n*=10), green tea (*n*=10), vitamin (*n*=6), sage tea (*n*=6), ginkgo, skrzyp polny, and sycamore leaves in order to highest to lower. Patients using herbal medications started by doctor recommendation were (1.7%), by publications (newspaper, Internet) (0.7%), by friend recommendation (1.2%), and by family members recommendation (0.2%).

2.7% of patients know the side effects of herbals, 92.5% of those do not know the side effects, and 4.8% of do not have any idea. Two patients from herbal users and 14 patients from nonherbal users know the side effects of herbal medications. Twelve of those who know the side effects learned this knowledge from the doctor. 11.3% of patients indicated that herbal medications should be stopped before surgery, 4.3% of patients indicated that herbal medications should not be stopped before surgery, and 84.3% of patients indicated that they have no idea. Those who say it should be stopped before surgery learned this information from the doctors. 57.8% of the cases using herbal have not operated before. 7.2% of those who had surgery stated this to the physician and 35% did not. While 35 cases of operation patients were questioned by the physician about herbal use, 228 cases were not questioned by the physician.

## 4. Discussion

According to the literature, the ratio of herbal consumption among patients varies between 4.8 and 32% [5, 10]. Our study showed that 14.5% of the presurgical patients are consuming herbal medications. This ratio is in agreement with the literature. On the other hand, patients are using these medications without the knowledge of their harmful effects and drug interactions. Also, they do not inform the anesthesiologist that they are using herbal medications preoperatively.

Skinner and Rangasami [11] reported self-administration of herbal medications is common in patients presenting for anaesthesia. The most commonly used medications are, in descending order, garlic, ginseng, ginkgo, St. John's wort, and *Echinacea*.

Onyeka et al. [8] showed that 40% of respondents reported the use of herbal medicine in the immediate perioperative period, and 87.5% did not inform their doctor of their herbal use.

İyilikçi et al. [12] determined the extent of herbal medicine use in children through preanesthetic evaluation. They reported that 32% of the children had a current or past history of ingestion of herbal medicines. The most commonly used herbals are garlic, green tea, and urtica drocia. So the importance of herbal medicines before anaesthesia and surgery is not only a problem in elderly, but also it is a problem in childhood.

The plants have been used for medical purposes since about 60,000 BC. Twenty-five percent of the drugs used today are isolated from plants and formated with chemical modifications. At present, a growing number of patients are suffering herbal medications such as *Echinacea*, ginger, *Ginkgo biloba*, ginseng, St. John's wort, valerian, *Ephedra*, kava, grapefruit juice, and turmeric [7].

The concept of complementary and alternative medicine has been used more frequently by the influence of press and Internet and nowadays found a wrong area of application especially in the treatment of cancer patients where conventional medicine is helpless [13]. Prevalence of herbal use was reported as 12.7% for *Echinacea*, 18% for *Efedra*, 7.9% for garlic, 2.6% for zincing, 8.6% for ginko, and 7.4% for ginseng in the preoperative period by Şencan et al. [13].

Unfortunately, the morbidity and mortality associated with the use of herbal drugs is increasing [7]. There have also been several case reports of unexpected surgical bleeding associated with garlic consumption [4, 7]. A 72-year-old man on no medication other than garlic tablets experienced bleeding after a transurethral resection of the prostate and required repeat cystoscopy and blood transfusion [14]. In another report, a healthy 32-year-old woman on no medication had significant bleeding after augmentation mammoplasty that required an evacuation of a haematoma. Further enquiries revealed heavy garlic intake [15], epidural hematoma due to excessive garlic consumption [7]. Several case reports of intracranial haemorrhage in patients using gingko have been published. Subarachnoid haemorrhage was reported in a 61-year-old man taking gingko tablets (40 mg tds) for 6 months. A 72-year-old woman suffered a subdural haematoma associated with ginkgo ingestion. There is a case report of bilateral subdural haematomas associated with chronic gingko ingestion in a 33-year-old woman. A 56-year-old man taking only gingko suffered a spontaneous right parietal intracerebral haematoma [3, 4, 7]. With ginkgo use, we may encounter not only with intracranial hemorrhage but also we may encounter with post surgical hemorrhage. Fessenden et al. [16] reported a 34-year-old healthy man who unexpectedly bled (haemoglobin concentration decreased from 16.5 g.dl to 5.4 g.dl) after a laparoscopic cholecystectomy. On further questioning, they revealed that he was taking two gingko tablets per day. Otherwise, renal failure on more than 100 patients with Chinese herbal tea mixtures had been reported [7].

Some of commonly used herbal medications are summarised in Table 1.

Pharmacodynamics and pharmacokinetics of majority of these medications have not been understand fully [1, 4, 6, 7, 11], so the American Society of Anesthesiologists (ASA) recommends discontinual of herbal medications 2-3 weeks before surgery [1, 4–7, 9, 11, 17–20]. Cheng et al. [1] advice to stop the herbal medicine 2 weeks before proceeding to anaesthesia and surgery. In March 1999, the American Society of Anaesthesiologists distributed a 2-minute video titled *Warning to Patients Taking Herbal Medications* to television stations nationwide to warn the American population of the potential problems of herbal medicines and surgery [4].

Taşpınar et al. [7] recommended 3 advices in their guideline on herbal medications published in 2011. (1) Bleeding tendency due to drug interactions and side effects is not be forgotten in patients at the perioperative period who have herbal medicine; (2) Although the duration of discontinuation of the drug before surgical treatment is different for each herbal medicine, they recommended to cut all of them 2 weeks before the surgery; (3) Closed compartment surgeries (e.g. intracranial) has high surgical risk.

In terms of patients' safety in perioperative care, it is crucial that patients must inform their physician about all substances they take. On the other hand, the physician's responsibility is to ask explicit questions to the patient about this topic in preoperative evaluation [5].

Patients still be taking herbal medications before surgery because either they are unaware of this recommendation or because they are presenting for emergency surgery [8, 11–13].

Physicians, irrespective of their speciality, should not underestimate the potential risks associated with the use of herbal medications [2, 19]. Especially, anesthesiologists and surgeons must have knowledge on commonly used herbal medicines and should be aware of the possible complications due to commonly used herbal medicines [7, 10, 21]. It is vital to have knowledge of herbal medications and their various interactions [3, 7, 19, 20]. Taşpınar et al. [7] studied awareness of medical specialist of herbal product use in preoperative stage. They reported that 57% of the participants claimed that usage of herbal medicine should be avoided in preoperative stage but only 8.6% of doctors managed to answer the question regarding the period of stopping the use of medicine accurately.

Doctors, especially anaesthetists, should be aware of the potential for interactions between conventional medications, herbal medicines, and anaesthesia and imbibe the culture of routine evaluation for herbal use, herbal pharmacovigilance, by instituting a herbal preoperative policy in their institutions and include herbal documentation in the anaesthetic charts, especially those for day case surgery. It would also be good practice to ask patients to discontinue all herbal medications, two weeks prior to the scheduled surgical procedure [8].

According to several reports, it is prevalent that presurgical patients are using herbal medications [9, 22]. In our hospital, there was no documentation of herbal medication on anaesthetic form. We believe that anaesthetist must document a full drug history including herbal medications in every patient. Documentation of the use of these medications is critical to determine the potential of drug or anesthetic interactions in perioperative period [8, 22].

## 5. Conclusion

All anesthesiologist should be aware of the effects of herbals on body functions and possible herbal-drug interactions. They must take care of such potentional perioperative complications with HDI. Hereby, they can choose the safest anaesthetic technique for their patient in the operating room [1, 6, 11–13, 21].

## Figures and Tables

**Table 1:** 

Common name	Pharmacological effects	Side effects	Recommeded cessation time
Garlic (*Allium sativum*)	Natural antibiotic, antiplatelet, antithrombolytic, lower lipid and cholesterol, reduced pulmonary and systemic resistance, potentiation of warfarin, risk of interaction with cardiovascular medications, MAO-I, hypoglycaemics [1, 3, 4, 6, 7, 9, 11, 13]	Epidural hematoma, postoperative bleeding, concerns for neuraxial blockage [4, 6, 7, 9, 11, 13], nausea, hypotension, allergy [3, 9]	Discontinued at least 7 days before surgery [3, 7, 9, 13]

*Echinacea*	Immunostimulatory effects, with long use may be immunosuppressive [1, 4, 6, 7, 9, 11, 13]	Hepatotoxic, with long use (8 weeks) it acts as immunosuppressant, reduced effectiveness of immunosuppressants, cause allergic and anaphylactic reactions [4, 6, 7, 9, 11, 13]	No data available, discontinue 2 weeks before surgery [9, 13]

Ephedra Ma huang	Sympathomimetic effects [1, 3, 4, 6, 7, 11, 13] weight loss, asthma treatment [9]	Autonomic side effects (tachycardia, hypertension, arrhythmia), myocardial infarction, stroke, seizure, renal stones, interacts with MAO-I, ergot alkaloids, halotan [1, 3, 4, 6, 7, 9, 11, 13]	Discontinued 24 hours preoperatively [3, 9]

*Ginkgo biloba*	Stabilise cognitive functions, vasoregulation, inhibition of thrombocyte activation factors, antagonize the activation of vitamin B6 [1, 3, 4, 6, 7, 9, 11, 13]	Spontaneous intracranial bleeding, hyphema, sedation, postoperative bleeding, decrease efficiency of anticonvulsants, may potentiate other platelet inhibitors [1, 3, 4, 6, 7, 9, 11, 13]	Discontinue 36 hours before surgery [7, 9, 13]

Ginseng *Panax ginseng*	Adaptogenic, lower blood glucose, inhibit platelet aggregation [1, 3, 4, 6, 7, 9, 13]	Hypoglycaemic effects (diabetics, posted surgery), interaction cardiovascular medications, digitalis and MAO-I [1, 3, 4, 6, 7, 9, 13]	Discontinue 24 hours before surgery [7, 13], discontinue 7 days before surgery [7, 9]

Kava *Piper methysticum*	Anxiolytic, sedative, antiepileptic, neuroprotective [3, 4, 6, 7, 9, 13]	Potentiate sedative effects of anaesthetic agents, hepatotoxic, GABA-R inhibition [3, 4, 6, 7, 9, 11, 13] cava dermopathy [3, 9]	Discontinue 24 hours before surgery [7, 9, 13]

St. John's wort *Hypericum perforatum*	Inhibited reuptake of serotonin, norepinenephrine, and dopamine by the neurons, induction of cytochrome P4503A4, P450 2C29 [1, 3, 4, 6, 7, 9, 11, 13]	Serotoninism, drug interaction with cyclosporine, digitalis, MAO-I, warfarin, oral contraceptives, throphylline, midazolam, lignocaine, sedative effect [1, 3, 4, 6, 7, 9, 11, 13]risk of organ rejection, increases uterine tone [3, 9]	Stopped 5 days before the surgery [3, 7, 9, 13]

Valerian *Valeriana officinalis*	Sedative, inhibited reuptake of GABA [3, 4, 6, 7, 9, 11]	High risk of potentiating of anesthetic and adjuvant drug actions, withdrawal-type syndrome with sudden abstinence [3, 4, 6, 7, 9, 11, 13]	No data available, discontinue 2 weeks before surgery [9]

Ginger *Zingiber officinale*	Useful in parturients who are suffering from hyperemesis, motion sickness, anti-inflamatuar, inhibited thromboxane synthetases activity [1, 4, 6, 7, 9, 13]	Possible mutagenesis, bleeding complications with warfarin, antiplatelet, risk of hyperglycaemia, abrupt discontinuation produce benzodiazepine withdrawal like symptoms [1, 4, 6, 7, 9, 13]	No data available, discontinue 2 weeks before surgery [9]

Green tea	Neurogenerative disease, anticancer, hypolipidemic [3]	Caffeine and tannins cause stimulation of CNS, source of vit. K, antioxidant. It may antagonise actions of warfarin, may cause arrhythmias, caution in renal and thyroid disease, insomnia [3]	No data available, discontinue 2 weeks before surgery

Grapefruit juice	Decreased atherosclerotic plaque formation and inhibit cancer cell proliferation [3, 4]	Inhibit CYP3A4 and alter the metabolism of various medications [3, 4]	No data available, discontinue 2 weeks before surgery
